# A network for swimming

**DOI:** 10.7554/eLife.28780

**Published:** 2017-07-05

**Authors:** Yee Lian Chew, William R Schafer

**Affiliations:** MRC Laboratory of Molecular Biology, University of Cambridge, Cambridge, United Kingdom; MRC Laboratory of Molecular Biology, University of Cambridge, Cambridge, United Kingdomwschafer@mrc-lmb.cam.ac.uk

**Keywords:** ciliary nerve, serotonin, zooplankton, connectomics, catecholamines, acetylcholine, *P. dumerilii*

## Abstract

A map of a neuronal circuit in a marine worm reveals how simple networks of neurons can control behavior.

**Related research article** Verasztó C, Ueda N, Bezares-Calderón LA, Panzera A, Williams EA, Shahidi R, Jékely G. 2017. Ciliomotor circuitry underlying whole-body coordination of ciliary activity in the *Platynereis* larva. *eLife* **5**:e26000. doi: 10.7554/eLife.26000

One of the fundamental aims of neuroscience is to understand how circuits of neurons interact to generate complex behavior. Toward that end, efforts are underway to generate complete maps of how all the neurons in a nervous system connect to each other. A complete human 'connectome' is many years away. Therefore, networks of neurons in simpler nervous systems, such as those from fruit flies and nematodes, are being mapped and analyzed as prototypes for understanding the network structure and circuit principles that may underlie bigger brains.

Now, in eLife, Gáspár Jékely and colleagues at the Max Planck Institute for Developmental Biology – including Csaba Verasztó as first author – report details of the connectome responsible for the locomotion of the larvae of a marine worm called *Platynereis dumerilii* (Verasztó et al., 2017).

The larvae of *P. dumerilii* swim by moving hair-like structures called cilia in a coordinated manner in response to visual and other sensory cues. These cilia are organized into several bands along the body of the animal known as the prototroch, metatroch and paratroch (Figure 1A). The prototroch sits between the head and the trunk, while the metatroch and three paratroch bands are spread along the trunk. Several patches of cilia are also found in the head of the larva, including on a specialized cell called the crescent cell.

**Figure 1.**
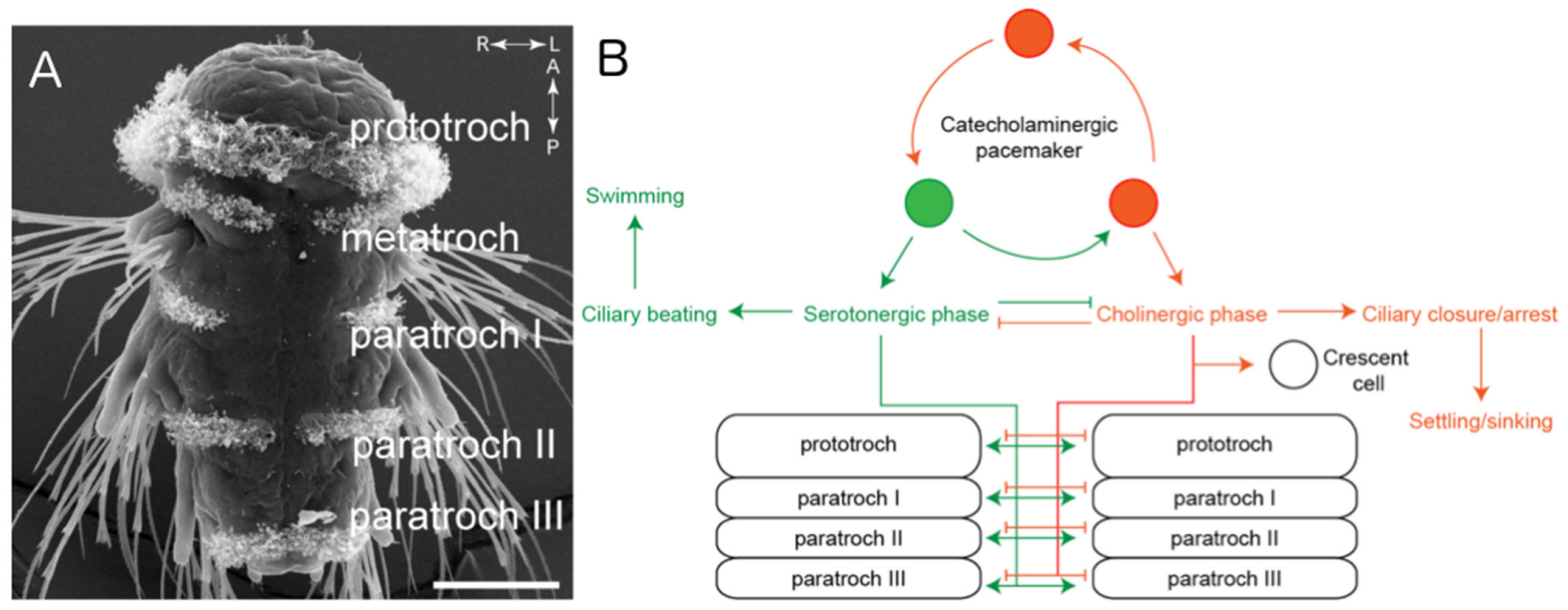
The ciliomotor circuitry of *Platynereis dumerilii*. (**A**) The larvae of *Platynereis dumerilii* swim by coordinating the beating of cilia on the surface of the body. The cilia are arranged into several bands known as the prototroch, metatroch and paratroch I, II and III. Scale bar, 50 μm. (**B**) Verasztó et al. mapped the connections between the three classes of motor neurons involved in swimming. The first class of neurons produce a neurotransmitter called acetylcholine and stop the cilia in the prototroch and the three bands of the paratroch beating during the cholinergic phase. These cholinergic neurons also connect to the crescent cell in the head, which has cilia that beat alternately to the cilia in the body. The second class of neurons produce a neurotransmitter called serotonin and make the cilia in the prototroch and paratroch beat faster during the serotonergic phase. The third class of neurons – known as catecholaminergic/peptidergic neurons – form a rhythmic pacemaker system that modulates cilia activity. Figure adapted from Verasztó et al., 2017.

The visual/motor system in the larvae contains only 71 cells (including the photoreceptors that collect visual cues, sensory neurons and the motor neurons that control the beating of the cilia). The wiring of the sensory layers of this system have been mapped previously by a technique called serial electron microscopy reconstruction (Randel et al., 2014), but the connections between the motor neurons and the ciliated cells had not been mapped.

Using neurochemical labeling techniques, Verasztó et al. mapped the neural inputs to the different bands of cilia, subdividing the neurons into three main classes based on the types of neurotransmitter molecules used to relay information between them. The first class (which rely on a neurotransmitter called acetylcholine) is composed of both sensory and motor neurons, including a neuron known as the MC neuron (which is connected to all the cells in the prototroch) and “Loop” neurons that communicate with all of the bands of cilia except the metatroch. Furthermore, these Loop neurons are the only input to the crescent cell.

The neurons in the second class – which produce a neurotransmitter called serotonin – include two “Ser-tr1” cells that connect to cells in the prototroch, metatroch and paratroch. Finally, the third class, known as catecholaminergic/peptidergic neurons, contains three cMN cells that produce different combinations of neurotransmitters, including dopamine, noradrenaline and neuropeptides. These three cells form connections amongst themselves, and also to the prototroch and the MC neuron.

Verasztó et al. then examined the roles these newly-mapped connections play in locomotion using neurochemical and live imaging approaches. This revealed that the MC neuron and the Loop neurons were active when the prototroch cells were active and the cilia were not beating. This suggests that these neurons stop all the cilia on the larva from beating. Consistent with this idea, using a drug to block acetylcholine receptors in the larvae caused the cilia to beat continuously.

The second class of neurons appear to play the opposite role in locomotion because treating the larvae with serotonin caused the cilia to beat faster and without stopping. Also, the Ser-tr1 cells became more active when the cilia in the prototroch band were beating more slowly, presumably to stimulate the cilia to beat faster. Lastly, the three cMN cells from the third class of neurons showed spontaneous rhythmic patterns of activity, with the activity of two of them increasing in synchrony with the activity of the cilia on the prototroch cells, whereas the activity of the third cMN cell was negatively correlated with this activity. Taken together, the results indicate that neurons producing different neurotransmitters are activated in alternating phases to control the beating of the cilia (Figure 1B).

This work demonstrates the power of connectomics to understand how neuronal activity modulates behavior in a simple organism. An interesting contrast can be made with *C. elegans,* a nematode that also has a simple connectome. While a map of the junctions between the neurons in *C. elegans* has been available for decades (White et al., 1986) and the pathways of dopamine, noradrenaline and serotonin activity are largely mapped (Bentley et al., 2016), its neuropeptide systems are extremely complicated and thus mostly uncharacterized. This is due to the worm neurons producing a vast number of different neuropeptides, many with uncharacterized activity, unidentified receptors, or unknown expression patterns.

On the other hand, all of the neuropeptides found in *Platynereis* have been identified and the other components of its neuropeptide systems are better understood. Thus, there is a real prospect of obtaining a complete connectome for *Platynereis* that could shed light on how neurotransmitters, which often signal between cells that are not directly connected by synapses, interact with the wired circuitry that makes up the connectome. Another difference between *Platynereis* and *C. elegans* is that the simplicity of the *C. elegans* nervous system probably evolved from a more complex nematode ancestor (Malakhov and Hope, 1994), whereas the *Platynereis* nervous system may more closely resemble that of a distant, more primitive ancestor (Jékely, 2011). If so, the structure of the *Platynereis* connectome may provide interesting insight into the origin of animal brains.
